# A cross‐sectional study comparing passive and eccentric modes of an isokinetic dynamometer to assess eccentric torque in trained athletes: Methodological considerations

**DOI:** 10.1002/ejsc.12248

**Published:** 2025-01-25

**Authors:** Anne Delextrat, Andreu Sastre‐Munar, Mark de Ste Croix, Gregory Walsh

**Affiliations:** ^1^ Department of Sport and Health Sciences and Social Work Oxford Brookes University Oxford UK; ^2^ Nursing and Physiotherapy Department University of the Balearic Islands Palma de Mallorca Spain; ^3^ School of Education and Science University of Gloucestershire Gloucester UK

**Keywords:** angle of peak torque, angle‐specific torque, peak torque, rate of torque development

## Abstract

Some technical limitations to using the eccentric mode to measure peak eccentric strength of the hamstrings (PTH_ecc_) were raised. PTH_ecc_ also has limited validity to predict performance or injury risk factor. Therefore, our aim was to compare PTH_ecc_ and other isokinetic variables tested in the eccentric and passive modes. Twenty male hockey players (20.2 ± 1.1 years; 179.7 ± 6.9 cm; 73.4 ± 7.1 kg and 12.2 ± 3.4% of body fat) performed maximal eccentric contractions of the hamstrings at 60°·s^−1^ (three repetitions) and 180°·s^−1^ (five repetitions) on both legs and using the eccentric mode and the passive mode (automatic movement of the lever arm) of the Biodex System 4 isokinetic dynamometer. The following variables were measured: PTH_ecc_, the angle of peak torque (APT,°), angle‐specific H_ecc_ torque at intervals of 10° and the rate of torque development (RTD) in the first 50 ms and the first 100 ms. The main results showed that compared to the eccentric mode, the passive mode led to a significantly greater PTH_ecc_ in the non‐dominant leg only and significantly smaller APT, RTD and angle‐specific H_ecc_ at angles close to knee flexion. In contrast, significantly greater angle‐specific H_ecc_ was observed in the passive mode at angles close to extension (10°–40°). This suggests that, although eccentric or concentric modes can be used to compare isokinetic data to existing literature, it is preferable to use the passive mode to assess peak torque or torque close to knee extension. The eccentric mode might be better to assess variables at the start of movement such as RTD.

## Highlights

- Assessing the eccentric strength of the hamstrings is classically done using the eccentric mode, which has some technical limitations. Although only a few studies use the passive mode for this, it seems more appropriate.
- The passive mode allowed participants to produce a significantly greater eccentric hamstring peak torque in the non‐dominant leg only, with an angle of peak torque (APT) closer to full knee extension, compared to the eccentric mode, and hence is better to use to measure maximal effort.
- In the passive mode, participants developed significantly greater eccentric hamstring torque at angles close to knee extension (10°–40°) compared to the eccentric mode. Consequently, the passive mode seems preferable to use to measure outcomes at these angles.
- The passive mode did not allow participants to produce a rate of torque development as high as in the eccentric mode. Although this variable is most commonly measured during isometric contractions, the eccentric mode could be more appropriate to use compared to the passive mode to measure variables at the start of the movement when eccentric contractions are examined.

## 1 INTRODUCTION

Isokinetic dynamometry (IKD) is widely used in sport and clinical settings to assess performance, test injury risk factors and rehabilitation outcomes (Almeida et al., 2023; Olmez et al., 2024; Yildiz et al., 2023). It is considered as the gold standard for strength testing (Santos et al., 2013) and can establish an individual's profile across various angular velocities, throughout the entire range of movement, and at varying modes of contraction (Ellenbecker & Davies, 2000; Schleichardt et al., 2021). The knee joint is the most commonly tested due to the major role of the muscles surrounding the knee in sport performance and its high risk of injury in athletes (i.e. Anterior Cruciate Ligament injury (ACLI), hamstring strain injury (HSI)), healthy/ageing individuals and clinical populations (i.e. osteoarthritis) (Aslan et al., 2020).

Hamstring eccentric torque is classically measured as an indicator of performance or thigh/knee injury risk using the eccentric mode on isokinetic dynamometers (Bourne et al., 2015). However, some authors suggested that using the passive mode might be better due to limitations of the eccentric mode (e.g. De Ste Croix et al., 2015) such as the initial contraction required to start the movement of the lever arm and the necessity to keep a minimal level of force throughout the movement. These could lead to stalling of the lever arm (if the athlete's torque falls below the required level), that could affect athletes' ability to cover the full range of motion (ROM), in particular at angles close to full leg extension, and result in torque spikes that can overestimate peak torque values and affect any data resulting from curve analysis (torque‐angle or torque‐time profile). In addition, the stalling and restarting is a safety issue, in particular when testing participants at relatively high angular velocities. In contrast, the movement of the lever arm in the passive mode starts automatically and is maintained throughout the range, hence eliminating these issues. Although these observations on the eccentric mode could have detrimental consequences on performance or risk factor evaluation and the passive mode could be a good alternative, there is currently no study comparing torque values obtained in both modes.

The main variables classically extracted from the IKD hamstring eccentric assessment to characterise the knee joint performance or its susceptibility to injury are peak torque (e.g. Bourne et al., 2015) and the rate of force (or torque) development (RFD, or RTD, Zebis et al., 2011). However, the relevance of peak torque was recently questioned, as hamstrings peak eccentric torque did not discriminate between playing levels in footballers (Eustace et al., 2020). In addition, some prospective studies and a meta‐analysis highlighted the limited predictive effect of this variable and the angle at which it is produced on HSI (Green et al., 2018; van Dyk et al., 2016, 2017). Considering torque production across the entire ROM could offer a more relevant and comprehensive assessment of the knee joint (De Ste Croix et al., 2015, 2018; Pieters et al., 2022; Sancese et al., 2023). For example, Naclerio et al. (2015) observed that hamstring eccentric torque significantly improved only at 30° and 20° from knee extension after six weeks of eccentric training, and suggested that these angle‐specific adaptations could help better tailor training programmes to specific athletic requirements. In the field of injury prevention, the torque produced at angles close to knee extension seem to be playing the most crucial role as HSI and ACLI occur at these angles (Yu & Garrett, 2007). Within this context, Lee et al. (2023) reported that compared to previously uninjured athletes, athletes with a previous ACLI showed similar hamstring eccentric peak torque, but significant deficits in hamstring eccentric torque at 30° and 20° from knee extension.

Therefore, the primary aim of this study was to compare peak eccentric strength of the hamstrings (PTH_ecc_) and other newly introduced isokinetic risk factors for injuries tested in the eccentric and passive modes in trained athletes. A secondary aim is to present these variables analysed in different ways reported in papers to allow for broader comparisons.

## 2 METHODS

### 2.1 Participants

Twenty male participants (20.2 ± 1.1 years; 179.7 ± 6.9 cm; 73.4 ± 7.1 kg and 12.2 ± 3.4% of body fat) were recruited from two University field hockey teams for this study. An a priori power analysis based on a study with a similar design suggested a sample size of 12 (alpha = 0.05; power = 0.80 and ƞp2 = 0.58) (Wiesinger et al., 2020). At the time of the study, the teams were involved in the British Universities and Colleges Sports National League and Midlands Tier Two. They had similar training loads, consisting in two 120 min field hockey practice sessions, one match and two 60 min strength and conditioning sessions weekly. Exclusion criteria were any current injury or a hamstring injury sustained within the past 12 months as well as any current medical treatment that could affect muscular performance. Participants gave written informed consent and the study was approved by the local University ethics committee in accordance with the principles set forth in the Helsinki declaration (University Research Ethics Committee, approval number 191305).

### 2.2 Procedures

Participants visited The University laboratory on one occasion only. After measuring their anthropometric characteristics (Height on a Seca stadiometer, Leicester, UK, and body mass and body fat on a Tanita BC 418MASegmental Body Composition Analyser, Tanita Corporation, Tokyo, Japan), they performed two different hamstring strength tests on an isokinetic dynamometer (Biodex system 4; Biodex, Shirley, NY). Isokinetic dynamometry was shown to provide a safe, reliable and objective evaluation of a large number of performance variables or injury risk factors and assessment of training benefits compared to other methods such as manual testing (Ellenbecker & Davies, 2000; Schleichardt et al., 2021).

### 2.3 Strength testing

After a 10 min standardised warm‐up on an ergocycle (Monark 874E; Monark, Varberg, Sweden) at 100 W with 6 s intermittent sprints in the last 4 min, participants completed isokinetic dynamometer strength testing (Biodex system 4; Biodex, Shirley, NY). They were seated on the dynamometer with their hips flexed at 90° according to guidelines previously described (Delextrat et al., 2020). Strength testing was performed using two modes, eccentric and passive. In the eccentric mode, the lever arm moves isokinetically at a pre‐set velocity (with the exception of acceleration and deceleration phases), with minimum and maximum torque thresholds. The minimum threshold requires a minimum eccentric force to be generated in order to move the lever arm. If the maximum torque threshold is achieved, the lever arm remains in a static hold position until the generated torque decreases. In the passive mode, the lever arm also moves isokinetically (with the exception of acceleration and deceleration phases), but the movement (at a pre‐set velocity) is generated by the machine irrespective of the torque applied by the participant, hence no minimum force is needed. Each test started with the knee flexed at 100° and ended with the leg extended (according to participants ROM, 0° being full knee extension), with manual resetting of these positions for the second leg tested. Participants were given similar verbal encouragement in all testing conditions to provide maximal effort. Testing started with a preliminary set to assess maximal concentric contractions of the quadriceps and hamstrings at 60°·s^−1^ (3 repetitions, preceded by 3 practice trials) and 180°·s^−1^ (5 repetitions, preceded by 5 practice trials) in order to establish individual torque limits for the eccentric mode (Hernandez et al., 2015). The strength tests were performed on both legs (the dominant leg was defined as the one with the greater peak torque achieved) and at two angular velocities, 60°·s^−1^ and 180°·s^−1^, with 2 min rest between modes and 5 min rest between legs. Three repetitions were performed at 60°·s^−1^ (preceded by 3 practice trials) and five repetitions at 180°·s^−1^ (preceded by 5 practice trials) as commonly used in the literature (e.g. Delextrat et al., 2010). The lower number of repetitions at 60°·s^−1^ compared to 180°·s^−1^ was chosen to avoid fatigue. During the practice trials, participants were instructed to increase the level of effort between the first (about 50% of their maximal effort) and last repetition (100% of maximal effort). The mode, leg and angular velocity conditions were randomised. The velocities chosen are characterised by the excellent test–retest reliability (Intraclass correlation coefficients of 0.95–0.98, Drouin et al., 2004).

### 2.4 Data analysis

Raw data were exported but not filtered (Bourne et al., 2015; Lehance et al., 2009; Opar et al., 2013). Two types of analysis were performed.

First, a number of commonly reported zero‐dimensional variables were extracted for each repetition of each leg in both modes and velocities. These variables included eccentric peak torque of the hamstrings (PTH_ecc_, N·m), peak torque asymmetry [(dominant‐non‐dominant)/dominant) × 100], in % and angle of peak torque (APT,°), defined as the angle at which PTH_ecc_ occurred. The angle‐specific H_ecc_ torque at 10°(H_ecc10_), 20°(H_ecc10_), 30°(H_ecc30_), 40°(H_ecc40_), 50°(H_ecc50_), 60°(H_ecc60_), 70°(H_ecc70_), 80°(H_ecc80_), 90°(H_ecc90_) and 100°(H_ecc100_) from full knee extension were also calculated. These were calculated as averages of values obtained at each position in windows of 10° (i.e. 0°–10°, 11°–20°, etc… de Ste Croix et al. (2018)). Rate of torque development in the first 50 ms and the first 100 ms (RTD_50_ and RTD_100,_ N·m·s^−1^) for H_ecc_ were calculated as the ratio between the change in torque and the corresponding change in time in the first 50 and 100 ms of contraction, respectively, as described by Zhang et al. (2021). The onset of contraction was defined as a torque value of 1% of the peak torque produced during the same contraction (Zhang et al., 2021). The time windows of 50 and 100 ms were chosen as the best compromise between the reliability and ecological validity (Krosshaug et al., 2007; Mentiplay et al., 2015). For each outcome measure, the best values out of the three or five contractions were then recorded for further analysis. It is the most commonly approach used in previous studies (Opar et al., 2013; de ste Croix et al., 2017; van Dyk et al., 2016; Zebis et al., 2011). In addition, the average of the two best contractions at 60°·s^−1^ and the three best contractions at 180°·s^−1^ were also calculated and results shown in the Appendix. It is the second most commonly used approach in the literature (Delextrat et al., 2020; Zhang et al., 2021).

The second type of analysis was based on statistical parametric mapping (SPM). For both modes at both speeds, raw unfiltered torque data were processed separately for each repetition. The whole contraction was analysed from the point where the torque crossed a threshold of 5% of the peak torque of that repetition to the point where torque dropped below the 5% threshold again or when the maximum angle of extension was reached if a second torque threshold crossing was not detected. For each repetition, torque data were interpolated to 101 data points to represent 0%–100% of the repetition, as the SPM analysis requires each signal to have the same number of data points.

### 2.5 Statistical analyses

All zero dimensional data were presented as mean ± standard deviation with 95% confidence interval. Statistical analyses were conducted using the Statistical Package for the Social Sciences (SPSS) statistical software (version 29.0, IBM Corp., Armonk, NY, USA). Initial tests were performed to check the normality of all variables using the Shapiro–Wilk test. Three‐way analyses of variance (ANOVA) with repeated measures and Bonferroni post hoc tests for multiple comparisons were performed to assess the effect of leg (dominant vs. non‐dominant), mode (eccentric vs. passive) and angular velocity (60°·s^−1^ vs. 180°·s^−1^) on peak H_ecc_, APT, RTD_50_ and RTD_100_ and the effect of mode (eccentric vs. passive), angular velocity (60°·s^−1^ vs. 180°·s^−1^) and angle (from 10 to 90) on angle‐specific H_ecc_ in each leg. In addition, a two‐way ANOVA with repeated measures and Bonferroni post hoc tests for multiple comparisons assessed the effect of mode (eccentric vs. passive) and angular velocity (60°·s^−1^ vs. 180°·s^−1^) on peak torque asymmetry. A *p*‐value < 0.05 was considered significant. Effect sizes were calculated as partial eta squared ƞp2 for the ANOVA and interpreted as no effect (0–0.05), minimum effect (0.05–0.26) and strong effect (0.26–0.64), whereas Cohen's *d* represented the effect size for post hoc tests and were interpreted as small (>0.2), medium (>0.5) and large (>0.8) (Cohen, 1988).

The statistical analysis based on SPM was used to analyse the difference between modes, speeds and legs across the whole torque signal to supplement the traditional approach of extracting zero‐dimensional metrics from torque profiles (Pieters et al., 2022). SPM computes the F value, SPM{F}, for each point on the trajectories, a critical F value is calculated using random field theory such that only 5% of a smooth random Gaussian trajectory would cross this critical F threshold. Suprathreshold clusters where the critical F rises above the critical F threshold correspond to periods in the trajectory where there are significant differences between factors. A 3‐way (condition × speed × leg) ANOVA was conducted using SPM for the whole contraction. All data processing was performed in MATLAB (Mathworks, Natick, MA, USA) and SPM analyses were performed using the open‐source package spm1D for MATLAB (version M.0.4.10; https://spm1d.org/).

## 3 RESULTS

### 3.1 PTH_ecc_, PTH_ecc_ asymmetry, APT, RTD_50_ and RTD_100_

The 3‐way ANOVA showed significant main effects of leg (F(1,19) = 7.479, *p* = 0.013 and ${ƞ}_{p}^{2}$ = 0.282) and mode (F(1,19) = 6.358, *p* = 0.021 and ${ƞ}_{p}^{2}$ = 0.251) as well as a significant interaction between the leg and mode (F(1,19) = 8.668, *p* = 0.008 and ${ƞ}_{p}^{2}$ = 0.313) on PTH_ecc_. The post hoc test revealed a significantly greater PTH_ecc_ in passive compared to eccentric modes in the non‐dominant leg (+13.0%, *d* = 0.70 and +4.2% and *d* = 0.19, respectively, at 60°·s^−1^ and 180°·s^−1^, *p* = 0.001 and Table 1), whereas no significant differences between modes were observed in the dominant leg (*p* = 0.755, Table 1).

**TABLE 1:** Mean ± standard deviation (95% confidence interval) for the best value of the peak eccentric torque of the hamstrings (PTH_ecc_), asymmetry, angle of peak torque and rate of toque development in the first 50 ms (RTD_50_) and the first 100 ms (RTD_100_) collected in the eccentric and passive modes in the dominant (D) and non‐dominant (ND) legs at 60°·s^−1^ (60) and 180°·s^−1^ (180).

	Dominant leg		Non‐dominant leg	
	Eccentric	Passive	Eccentric	Passive
PTH_ecc_ 60 (N·m)	182 ± 32 (168–197)	184 ± 33 (169–200)	161 ± 31 (147–175)	182 ± 29^a^ (168–195)
PTH_ecc_ 180 (N·m)	186 ± 36 (169–202)	182 ± 36 (165–198)	168 ± 42 (149–188)	175 ± 32^a^ (160–190)
Asymmetry 60 (%)	11.7 (8.3‐15.1)	0.6 (‐9.3‐8.0)^a^		
Asymmetry 180 (%)	9.7 (2.9‐16.4)	2.2 (‐5.1‐9.6)^a^		
APT 60 (°)	19.7 ± 10.8 (14.7‐24.8)	14.2 ± 8.1^a^ (10.4‐18.0)	21.8 ± 13.3 (15.5‐28.0)	18.2 ± 13.2^a^ (12.0‐24.3)
APT 180 (°)	17.0 ± 11.8 (11.4‐22.5)	14.1 ± 8.6^a^ (10.1‐18.1)	18.8 ± 10.2 (14.0‐23.6)	16.5 ± 10.7^a^ (11.5‐21.5)
RTD_50_ 60 (N·m·s^−1^)	683 ± 393 (499–867)	459 ± 171^a^ (379–539)	767 ± 435 (564–971)	489 ± 264^a^ (365–612)
RTD_50_ 180 (N·m·s^−1^)	1226 ± 427^b^ (1026–1426)	1168 ± 420^a^ ^,^ ^b^ (971–1364)	1251 ± 412^a^ ^,^ ^b^ (1058–1444)	1163 ± 499^a^ ^,^ ^b^ (929–1396)
RTD_100_ 60 (N·m·s^−1^)	446 ± 214^a^ (346–546)	339 ± 119^a^ (283–395)	503 ± 297 (364–642)	355 ± 184^a^ (269–441)
RTD_100_ 180 (N·m·s^−1^)	617 ± 196^b^ (526–709)	587 ± 155^a^ ^,^ ^b^ (514–660)	579 ± 161^b^ (504–655)	572 ± 172^a^ ^,^ ^b^ (491–652)

^a^ Significantly different from the eccentric mode, *p* < 0.05.

^b^ Significantly different from 60°·s^−1^., *p* < 0.05.

A significant effect of mode only was shown on PTH_ecc_ asymmetry (F(1,19) = 10.634, *p* = 0.005 and ${ƞ}_{p}^{2}$ = 0.863), with the passive mode characterised by significantly lower asymmetry compared to the eccentric mode (*d* = 0.76 and *d* = 0.46, respectively, at 60°·s^−1^ and 180°·s^−1^, *p* = 0.005 and Table 1),

The statistical analysis showed a significant effect of the testing mode only on APT for H_ecc_ (F1,19) = 4.846, *p* = 0.040 and ${ƞ}_{p}^{2}$ = 0.230). The passive mode was characterised by significantly smaller values (i.e. closer to full knee extension) compared to the eccentric mode (−27.9%, *d* = 0.58 and −17.1% and *d* = 0.28, respectively, at 60°·s^−1^ and 180°·s^−1^ in the dominant leg and −16.5%, *d* = 0.27 and −12.2% and *d* = 0.23, respectively, at 60°·s^−1^ and 180°·s^−1^ in the non‐dominant leg *p* = 0.001 and Table 1).

Regarding the RTD, there was a significant effect of velocity (F(1,19) = 119.8, *p* < 0.001 and ${ƞ}_{p}^{2}$ = 0.863) and mode (F(1,19) = 12.581, *p* = 0.002 and ${ƞ}_{p}^{2}$ = 0.398) on RTD_50_. Post hoc analyses showed significantly greater values at 180°·s^−1^ compared to 60°·s^−1^ (+44.3%, *d* = 1.32 and + 60.7% and *d* = 2.21, respectively, in the eccentric and passive modes in the dominant leg and +38.7%, *d* = 1.14 and +58.0% and *d* = 1.69, respectively, in the eccentric and passive modes in the non‐dominant leg *p* = 0.001 and Table 1). In addition, the passive mode led to significantly smaller RTD_50_ compared to the eccentric mode (−32.8%, *d* = 0.74 and −4.7% and *d* = 0.14, respectively, at 60°·s^−1^ and 180°·s^−1^ in the dominant leg and −36.2%, *d* = 0.77 and −7.0% and *d* = 0.19, respectively, at 60°·s^−1^ and 180°·s^−1^ in the non‐dominant leg, *p* = 0.002 and Table 1). Similar results were observed on RTD_100_. The 3‐way ANOVA showed a significant effect of velocity (F(1,19) = 82.770, *p* < 0.001 and ${ƞ}_{p}^{2}$ = 0.813) and mode (F(1,19) = 7.137, *p* = 0.015) and ${ƞ}_{p}^{2}$ = 0.273) on RTD_100_. Post hoc analyses showed significantly greater values at 180°·s^−1^ compared to 60°·s^−1^ (+27.7%, *d* = 0.83 and + 42.2% and *d* = 1.79, respectively, in the eccentric and passive modes in the dominant leg and +13.1%, *d* = 0.32 and + 37.9% and *d* = 1.22, respectively, in the eccentric and passive modes in the non‐dominant leg *p* = 0.001 and Table 1). In addition, the passive mode led to significantly smaller RTD_100_ compared to the eccentric mode (−24.0%, *d* = 0.62 and −5.8% and *d* = 0.17, respectively, at 60°·s^−1^ and 180°·s^−1^ in the dominant leg and −29.4%, *d* = 0.60 and −1.2%and *d* = 0.04, respectively, at 60°·s^−1^ and 180°·s^−1^ in the non‐dominant leg, *p* = 0.015 and Table 1).

### 3.2 Angle‐specific H_ecc_

The statistical analysis showed a significant effect of angle (F(9,144) = 118.644 and *p* < 0.001) and interaction between the mode and angle (F(9,144) = 19.804 and *p* < 0.001) on H_ecc_ in the non‐dominant leg (Table 2). Post hoc analyses showed significantly greater values in the eccentric compared to passive modes at 100° (+43.2%, *d* = 1.49 and *p* < 0.001), 90° (+25.6%, *d* = 0.97 and *p* < 0.001) and 80° (+10.3%, *d* = 0.50 and *p* = 0.038). In contrast, significantly greater values were reached in the passive compared to eccentric mode at 40° (+8.9%, *d* = 0.43 and *p* = 0.015), 30° (+13.8%, *d* = 0.61 and *p* < 0.001), 20° (+13.8%, *d* = 0.50 and *p* = 0.006) and 10° (+14.1%, *d* = 0.46 and *p* = 0.014).

**TABLE 2:** Mean ± standard deviation (95% confidence interval) for the best value of angle‐specific eccentric torque of the hamstrings (H_ecc_) collected in the eccentric and passive modes in the non‐dominant (ND) legs at 60°·s^−1^ (60) and 180°·s^−1^ (180).

	60		180	
	Eccentric	Passive	Eccentric	Passive
H_ecc100_ (N·m)	46 ± 12 (39–52)	22 ± 15^a^ (14–30)	42 ± 14 (35–48)	27 ± 14^a^ (20–33)
H_ecc90_ (N·m)	77 ± 19 (67–87)	56 ± 22^a^ (45–67)	81 ± 24 (70–92)	59 ± 18^a^ (58‐49)
H_ecc80_ (N·m)	95 ± 22 (84–106)	85 ± 25^a^ (72–98)	98 ± 17 (90–106)	89 ± 18^a^ (81–97)
H_ecc70_ (N·m)	114 ± 24 (102–126)	108 ± 26 (95–121)	108 ± 23 (97–119)	111 ± 19 (102–120)
H_ecc60_ (N·m)	128 ± 24 (115–140)	128 ± 25 (116–141)	118 ± 29 (105–132)	129 ± 20 (118–139)
H_ecc50_ (N·m)	138 ± 28 (124–153)	145 ± 24 (132–157)	129 ± 37 (111–144)	143 ± 21 (132–153)
H_ecc40_ (N·m)	149 ± 30 (133–164)	158 ± 25^a^ (145–171)	137 ± 43 (117–157)	156 ± 25^a^ (142–163)
H_ecc30_ (N·m)	144 ± 36 (126–163)	169 ± 31^a^ (152–185)	144 ± 51 (121–167)	166 ± 29^a^ (151–179)
H_ecc20_ (N·m)	143 ± 48 (118–167)	169 ± 41^a^ (148–190)	145 ± 55 (119–169)	165 ± 39^a^ (146–182)
H_ecc10_ (N·m)	127 ± 45 (104–151)	156 ± 48^a^ (131–181)	129 ± 56 (103–153)	142 ± 40^a^ (123–160)

^a^ Significantly different from the eccentric mode, *p* < 0.05.

In the dominant leg, a significant effect of angle only was observed (F(9,144) = 2.7, *p* = 0.006 and Table 3).

**TABLE 3:** Mean ± standard deviation (95% confidence interval) for the best value of angle‐specific eccentric torque of the hamstrings (H_ecc_) collected in the eccentric and passive modes in the dominant (D) legs at 60°·s^−1^ (60) and 180°·s^−1^ (180).

	60		180	
	Eccentric	Passive	Eccentric	Passive
H_ecc100_ (N·m)	58 ± 16 (48–67)	26 ± 17 (15–37)	48 ± 13 (40–55)	30 ± 16 (18–39)
H_ecc90_ (N·m)	85 ± 19^a^ (71–96)	60 ± 20^a^ (49–72)	91 ± 18^a^ (80–103)	63 ± 19^a^ (51–75)
H_ecc80_ (N·m)	105 ± 22^a^ (89–118)	87 ± 20^a^ (76–99)	106 ± 13^a^ (97–115)	93 ± 20^a^ (80–105)
H_ecc70_ (N·m)	120 ± 24^a^ (105–135)	109 ± 22^a^ (97–123)	117 ± 17^a^ (106–128)	115 ± 23^a^ (101–129)
H_ecc60_ (N·m)	137 ± 256 (122–152)	130 ± 22^a^ (118–143)	131 ± 8^a^ (119–142)	134 ± 25^a^ (118–149)
H_ecc50_ (N·m)	153 ± 25^b^ (138–168)	146 ± 23^b^ (134–159)	144 ± 24^b^ (127–158)	147 ± 26^b^ (131–162)
H_ecc40_ (N·m)	166 ± 25^b^ (152–180)	160 ± 23^b^ (153–179)	156 ± 26^b^ (143–179)	159 ± 28^b^ (142–176)
H_ecc30_ (N·m)	173 ± 30^b^ (158–189)	171 ± 29^b^ (159–193)	163 ± 30^b^ (127–182)	170 ± 31^b^ (151–188)
H_ecc20_ (N·m)	173 ± 37 (152–193)	177 ± 42 (151–204)	159 ± 44 (131–183)	165 ± 42 (139–190)
H_ecc10_ (N·m)	155 ± 40 (134–176)	166 ± 43 (137–195)	143 ± 40 (118–167)	160 ± 42 (135–195)

^a^ Significantly different from all angles, except 10° and 100°, *p* < 0.05.

^b^ Significantly different from all angles, except 10°, 20° and 100°, *p* < 0.05.

### 3.3 Whole contraction SPM

There was a significant effect (*p* < 0.001) of mode from 0% to 51% of the contraction; torque was greater in the eccentric mode compared to the passive mode. There was also a significant effect (*p* < 0.001) of velocity for the whole contraction (0%–100%) with torque greater at 60°/s compared to 180°/s. Additionally, a significant two‐way mode*velocity interaction (*p* = 0.001) was present with the difference in torque between velocities greater in the eccentric mode compared to the passive mode. There was no main effect of leg, or leg*condition, leg*velocity or leg*condition*velocity interaction effects.

**FIGURE 1:**
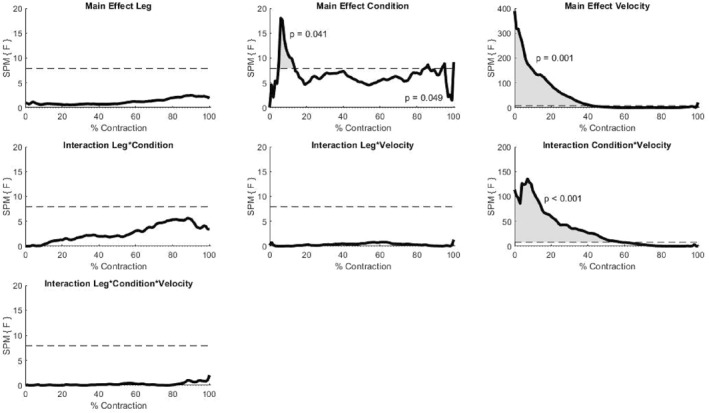
SPM{F} trajectories for the main effects of the leg (top left), mode condition (top middle) and velocity (top right); the interaction effects of leg*condition (middle left), leg*velocity (middle middle), condition*velocity (middle right) and leg*condition*velocity. The critical F for all effects is indicated by the horizontal dashed line and was *F* = 8.76. Periods of the trajectory where there was a significant effect (i.e. the F value rose above the critical F) are shaded great with the corresponding *p*‐value for that suprathreshold cluster presented next to this period on the graph. As such, it can be seen that there was a significant effect of the mode condition, velocity and condition*velocity interaction.

**FIGURE 2:**
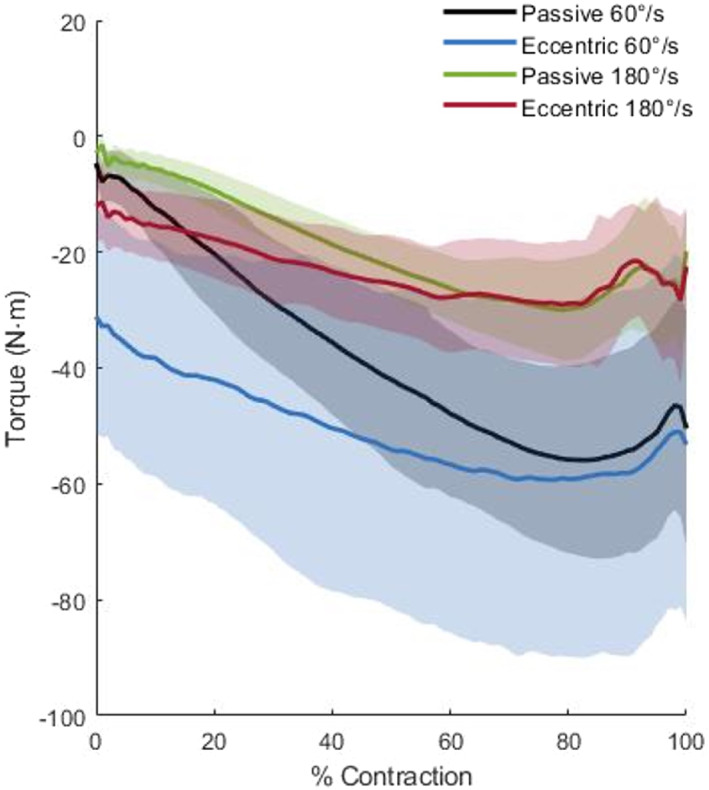
Mean torque with standard deviation clouds for the passive and eccentric modes at 60°·s^−1^ and 180°·s^−1^. As there were no significant effects of leg or interactions including the leg factor, data are presented as the combined mean of dominant and non‐dominant legs.

SPM results for all effects can be found in Figure 1. As there were no significant effects of leg or interactions including the leg factor, for ease of interpretation of plots of torque data, the data presented are combined dominant and non‐dominant leg data for each condition and velocity (Figure 2).

## 4 DISCUSSION

The main results of the present study showed that, considering analyses based on the best contraction, the passive mode led to a significantly greater PTH_ecc_ in the non‐dominant leg only and significantly smaller PTH_ecc_ asymmetry, APT and RTD compared to the eccentric mode. In addition, significantly greater torque values were observed in the eccentric compared to the passive mode at the start of the movement (first 51% of the movement or knee flexion angles of 80°–100°), whereas in contrast, significantly greater torque values were observed in the passive compared to eccentric mode at angles close to extension (10°–40°). Finally, when considering an analysis based on the average of the best two or three contractions, similar results to the analysis based on the best contraction were found, except no significant variation in APT across modes, angular velocities or legs, and significantly greater H_ecc_ in the eccentric compared to passive modes at angles of 100°, 90°, 80° and 70° in the dominant leg. These results indicate that the isokinetic variables commonly collected as indicators of athletes' performance or risk to develop a knee or thigh injury vary depending on the mode used and where in the range of movement torque is assessed. Although this might not seem like a major issue when using repeated measures with the same mode (where a potential “error” is repeated), such as testing performance before and after a fatigue protocol, or when evaluating the effectiveness of training programme, it can be more problematic in cross‐sectional designs (i.e. determination of maximal performance to establish normative data), in particular if comparing studies using different modes of assessment.

Peak isokinetic eccentric hamstring strength has been measured in many previous studies as an indicator of strength performance (Alhammoud et al., 2019; Eustace et al., 2020) or risk factor for HSI and ACLI (Bourne et al., 2015; Myer et al., 2009; Wild et al., 2013). Our results showed a significant leg*mode interaction on PTH_ecc_, with no significant difference between modes observed on PTH_ecc_ in the dominant leg, whereas a significantly greater PTH_ecc_ (+4.2% with a trivial effect size at 180°·s^−1^ and +13% with a medium effect size at 60°·s^−1^) was reached in the passive compared to eccentric mode in the non‐dominant leg. The lower peak torque in the eccentric mode in the non‐dominant leg could be partly explained by the fact that this mode requires participants to exert at least 10% of a present torque limit to move the dynamometer shaft. Although this torque limit was individually set‐up, some of the participants of the present study could not maintain a sufficient level of torque throughout the full ROM, in particular with their weaker (non‐dominant) leg, hence stalling the dynamometer shaft. These stops and restarts could influence the optimal motor unit recruitment pattern required to produce maximal torque, create local fatigue if this occurs frequently over several repetitions and/or make the movement less smooth. Although these observations are not measured scientific evidence, they could be backed up by the findings of some authors that the non‐dominant leg does not perform as well as the dominant leg in other hamstring eccentric exercises such as the Nordic hamstring exercise (Clark et al., 2005; Mendiguchia et al., 2013). In contrast, during isokinetic eccentric exercises where the lever arm is moving for them (passive mode), participants may not experience these stops and restarts potentially leading to exert a greater effort on the non‐dominant side. This is one of the reasons why many authors using isokinetic testing in the eccentric mode extract torque‐angle profiles using custom algorithms in order to filter the signal and eliminate movement artifacts (e.g. Delextrat et al., 2020). Another possible explanation for the lower PTH_ecc_ achieved in the eccentric mode is a lack of familiarisation. Indeed, Nugent et al. (2015) showed that one familiarisation bout is usually enough for the knee extensors, whereas movements involving the knee flexors required more familiarisation. Familiarisation with a movement is known to increase force production by decreasing the level of co‐contraction of the antagonist muscle (Carolan & Cafarelli, 1992). Therefore, we could hypothesise that our participants, similarly to those of many studies using limited familiarisation, may not have reached their maximal level of strength in the eccentric mode. These observations suggest that the passive mode might be appropriate to measure peak hamstring eccentric torque to ensure the achievement of maximal performance, especially if bilateral differences in strength are to be investigated within and between participants.

The lower PTH_ecc_ and greater asymmetry observed in the eccentric versus passive mode in the non‐dominant leg in the present study could have some implications on data recorded in experimental trials. Indeed, eccentric peak torque values of the hamstrings have been commonly used to compare the dominant and non‐dominant legs, with some thresholds identified to prescribe unilateral strengthening (Bourne et al., 2015; Fousekis et al., 2011). However, one could question the validity of these values, since the lower PTH_ecc_ observed in the non‐dominant leg is likely to have been underestimated in the eccentric mode similarly to the present study. Had the passive mode been used, it is possible that lower inter‐limb asymmetry could have been reported.

The results of the present study showed that the passive mode led to significantly greater torque at angles close to knee extension (10°–40°) compared to the eccentric mode. This could have crucial implications for training prescription/injury risk identification (ACLI and HSI are known to occur at these angles, Yu and Garrett (2007)) when comparing findings between studies if they used different modes. For example, contrasting results were reported on the benefits of the Nordic hamstring exercise on H_ecc_ measured at angles close to knee extension, with some authors reporting no benefits (Delextrat et al., 2020), whereas others found significant improvements (Naclerio et al., 2015; Sancese et al., 2023). It is difficult to assess whether these contrasting results are due to differences between training programmes in these studies or the lower capacity of participants to exert torque near knee extension in the eccentric mode in some studies (the eccentric mode for Delextrat et al. (2020) and Naclerio et al. (2015) versus the passive mode for Sancese et al. (2023).

In contrast with the greater torque developed towards knee extension in the passive mode, we observed significantly greater torque values in the eccentric compared to passive modes at angles close to knee flexion (80–100) corresponding to the start of the movement. These differences could be explained by the pre‐activation of the hamstrings in the eccentric mode due to the necessity to isometrically contract the hamstrings at the start of the movement against a pre‐set resistance to initiate the movement of the dynamometer shaft. No isometric contraction was needed in the passive mode as the movement was automatically initiated by the dynamometer. The process of pre‐activation corresponds to the various changes observed in a muscle when its contraction is preceded by a previous contraction, resulting in a greater force development during the second contraction, compared to a single contraction (Bobbert & Casius, 2005). Various mechanisms could be responsible for these adaptations including the recruitment of a larger number of motor units, their better synchronisation or enhanced firing rate as well as a calcium‐regulated increase in stiffness induced by the previous contraction (Schaefer & Bittmann, 2019). These adaptations are not mode‐specific, as previous studies have show, for example, that a greater concentric torque of the quadriceps was achieved when it was preceded by an eccentric or isometric contraction of this muscle group (Fukutani et al., 2016), and a greater eccentric torque of the quadriceps was developed after they performed an isometric contraction (Schaefer & Bittmann, 2019), following a sequence similar to the present study.

It seems that the benefits of pre‐activation are more important at the start of a movement compared to the end. Indeed, Fukutani et al. (2016) recorded the concentric torque produced by the quadriceps on an isokinetic dynamometer at 180°·s^−1^ within a ROM from 80 to 140 (180 being full extension) when it was preceded by no contraction, an isometric contraction of the quadriceps or an eccentric contraction of the quadriceps in 12 recreationally trained men. They showed a greater torque in the two conditions preceded by a contraction compared to the control, but more interestingly, these improvements were significant only at the start of the movement (85°) and did not remain after an angle of 105°. Although these results were obtained on a different muscle group and contraction mode, they seem to parallel the trend observed in the eccentric torque of the hamstring in the present study.

Another isokinetic variable classically collected at the start of the movement is RFD or RTD. Although RFD variables are typically measured in isometric conditions to control for the confounding influence of joint angle and angular velocity changes (Maffiuletti et al., 2016), some authors have measures RTD using concentric or eccentric contractions for several reasons, such as ecological validity (Alhammoud et al., 2018; Zhang et al., 2021), or to focus on mechanistic or aetiological aspects of eccentric contractions in specific populations (Opar et al., 2013). For example, Zhang et al. (2021) measured RTD during hamstring eccentric contractions because the capacity of the hamstring to quickly produce eccentric torque has been associated with ACLI known to occur within 0–61 ms of initial contact with the ground (Bates et al., 2020). The greater RTD observed in the present study in the eccentric mode suggests that this mode should be used rather than the passive mode to assess this variable where eccentric contractions are investigated. In addition, this mode reproduces more closely eccentric contractions occurring in sport situations, where muscle contractions rarely occur in an isolated manner, but instead pre‐activation often takes place.

A secondary aim of the present study was to compare the effects of different calculation methods for hamstring eccentric torque variables (i.e. best value (de Ste Croix et al., 2017; Opar et al., 2013; van Dyk et al., 2016; Zebis et al., 2011) and average of two or three best contractions (Delextrat et al., 2020; Zhang et al., 2021). Our findings show that there are only a couple of differences between the results obtained by both approaches, suggesting that our results can be applied to both methods for most variables (PTH_ecc_, RTD, angle‐specific torque measured in the non‐dominant leg). However, although the passive mode led to significantly smaller APT than the eccentric mode when the best contraction was considered, no significant differences were observed with average values. It is difficult to pinpoint the main reason for these differences, as taking the best value is generally more likely to show differences between variables, especially at greater, less reliable angular velocities (Drouin et al., 2004). However, we observed the opposite on angle‐specific values, with significant differences (greater H_ecc_) at 100°, 90°, 80° and 70° in the eccentric versus passive mode in the dominant leg with average values, and no significant difference for best values. These results need to be taken into account when interpreting isokinetic data and comparing studies using different modes. Recently, some authors have approached isokinetic torque calculations by performing analyses (SPM) of entire torque‐angle or torque‐time curves. For example, Pieters et al. (2022) identified that football players with a previous HSI were significantly stronger in concentric hamstring and quadriceps strength, eccentric hamstring strength and concentric hip extensor torques, but significantly weaker concentric in hip flexion torque performance throughout the entire ROM. These authors tested eccentric torque using the eccentric mode on an isokinetic dynamometer, and it would be interesting to find out if using the passive mode would lead to similar results, given the difference that we observed in the first half of the movement.

The present study has some limitations including the characteristics of our participants, familiarisation, data processing and sample size. Indeed, hockey players have relatively large strength asymmetry between legs compared to other team sport players (Delextrat et al., 2020), which could have influenced the difference between legs. In addition, although familiarisation with the protocol was included in our study, greater familiarisation could have been beneficial. We also did not perform any data processing (filtering etc…) in order to capture the real differences between modes more objectively. This could slightly influence the application of our findings, as some authors work on the raw data, similarly to us, whereas others apply various filters to their data. Finally, although this study was adequately powered, some of our effect sizes are small and hence should be interpreted with caution.

In conclusion, our findings showed that testing hamstring eccentric strength in the passive mode leads to a significantly greater PTH_ecc_ in the non‐dominant leg and significantly smaller APT, RTD and angle‐specific torque at the start of the movement (first 51% of the movement or knee flexion angles of 70°–100°), whereas the torque developed towards the end of range (10°–40°) were significantly greater. This suggests that it might be preferable to use the passive mode as an objective method to assess peak torque or torque close to knee extension. In contrast, the eccentric mode might be better to assess variables at the start of the movement, such as RTD, even if this variable is more commonly measured isometrically. Finally, authors should be aware of these differences when characterising the torque–angle relationship throughout the ROM or calculating variables representing the entire curve such as the work produced. Further studies should investigate the effects of other factors such as neuromuscular fatigue on torque–angle relationship measured in the passive mode.

## CONFLICT OF INTEREST STATEMENT

The authors declare no conflicts of interest.

## Data Availability

Anonymised data for this research is available online on the University Research And Digital Assets Repository, following this link: https://radar.brookes.ac.uk/radar/items/0b4b4813-a05a-4a75-91a5-527e1773bfb6/1/.

## PTH_ecc_, APT, RTD_50_ and RTD_100_ (Average of best two/three contractions)

These results are presented in Table A1. They showed the same effects as the ones observed for the best contraction results, except that there was no significant effect of any independent variable tested on APT based on an average value of the best two/three contractions (*p* > 0.05).

**TABLE A1:** Mean ± standard deviation (95% confidence interval) for the average of the two/three best values of the peak eccentric torque of the hamstrings (PTH_ecc_), asymmetry, angle of peak torque and rate of toque development in the first 50 ms (RTD_50_) and the first 100 ms (RTD_100_) collected in the eccentric and passive modes in the dominant (D) and non‐dominant (ND) legs at 60°·s^−1^ (60) and 180°·s^−1^ (180).

	Dominant leg		Non‐dominant leg	
	Eccentric	Passive	Eccentric	Passive
PTH_ecc_ 60 (N·m)	176 ± 31 (162–191)	178 ± 32 (163–192)	153 ± 32 (138–169)	176 ± 28^a^ (163–189)
PTH_ecc_ 180 (N·m)	176 ± 35 (159–192)	175 ± 36 (158–192)	157 ± 43 (136–177)	167 ± 32^a^ (152–182)
Asymmetry 60 (%)	13.7 (9.7‐17.7)	0.9 (‐8.0‐9.8)^a^		
Asymmetry 180 (%)	9.7 (2.9‐16.4)	3.7 (‐4.5‐12.0)^a^		
APT 60 (°)	19.3 ± 9.5 (14.9–23.7)	17.7 ± 8.9 (13.5–21.8)	24.5 ± 12.1 (18.8–30.2)	19.0 ± 13.1 (12.9–25.1)
APT 180 (°)	17.8 ± 9.0 (13.6–22.0)	17.7 ± 12.4 (11.9–23.5)	21.1 ± 10.3 (16.3–25.9)	17.8 ± 8.1 (14.0–21.6)
RTD_50_ 60 (N·m·s^−1^)	495 ± 277 (365–624)	367 ± 103^a^ (318–415)	498 ± 285 (364–631)	383 ± 177^a^ (300–466)
RTD_50_ 180 (N·m·s^−1^)	960 ± 366^b^ (788–1131)	863 ± 350^a^ ^,^ ^b^ (699–1027)	988 ± 327^a^ ^,^ ^b^ (835–978)	956 ± 390^a^ ^,^ ^b^ (773–1139)
RTD_100_ 60 (N·m·s^−1^)	331 ± 157^a^ (258–404)	279 ± 86^a^ (239–319)	352 ± 185 (266–439)	289 ± 130^a^ (228–350)
RTD_100_ 180 (N·m·s^−1^)	502 ± 126^b^ (442–561)	469 ± 98^a^ ^,^ ^b^ (424–515)	491 ± 138^b^ (427–556)	472 ± 121^a^ ^,^ ^b^ (415–528)

^a^ Significantly different from the eccentric mode, *p* < 0.05.

^b^ Significantly different from 60°·s^−1^, *p* < 0.05.

## Angle‐specific H_ecc_ (Average of best two/three contractions)

These results are presented in Tables A2 and A3. The statistical analysis showed the same ANOVA effects as for the results presented for the best contraction for the non‐dominant leg as well as the same significant pairwise differences. In the dominant leg, the statistical analysis showed a significant effect of mode (F(1,19) = 7.659, *p* = 0.017, ${ƞ}_{p}^{2}$ = 0.390), angle (F(9,144) = 94.444, *p* < 0.001, ${ƞ}_{p}^{2}$ = 0.887), velocity*angle interaction (F(9,144) = 3.835, *p* = 0.025 and ${ƞ}_{p}^{2}$ = 0.242) and mode*angle interaction (F(9,144) = 8.317, *p* = 0.002 and ${ƞ}_{p}^{2}$ = 0.409). Post hoc analyses showed significantly greater H_ecc_ in the eccentric compared to passive modes at 100° (+52.9%, *d* = 1.93 and *p* < 0.001), 90° (+36.2%, *d* = 1.51 and *p* < 0.001), 80° (+16.7%, *d* = 0.65 and *p* = 0.004) and 70° (+8.2%, *d* = 0.40 and *p* = 0.036).

**TABLE A2:** Mean ± standard deviation (95% confidence interval) for the average of the best two/three values of angle‐specific eccentric torque of the hamstrings (H_ecc_) collected in the eccentric and passive modes in the non‐dominant (ND) legs at 60°·s^−1^ (60) and 180°·s^−1^ (180).

	60		180	
	Eccentric	Passive	Eccentric	Passive
H_ecc100_ (N·m)	38 ± 10 (33–44)	18 ± 12^a^ (12–24)	40 ± 16 (31–48)	19 ± 10^a^ (13–24)
H_ecc90_ (N·m)	70 ± 19 (61–81)	46 ± 20^a^ (35–57)	76 ± 31 (60–92)	44 ± 16^a^ (35–52)
H_ecc80_ (N·m)	88 ± 21 (77–100)	78 ± 26^a^ (64–92)	96 ± 16 (87–104)	74 ± 17^a^ (65–82)
H_ecc70_ (N·m)	107 ± 24^b^ (94–121)	103 ± 27^b^ (88–117)	105 ± 25^b^ (92–118)	99 ± 17^b^ (90–108)
H_ecc60_ (N·m)	123 ± 25^c^ (109–137)	123 ± 24^c^ (110–136)	118 ± 28^c^ (102–133)	120 ± 18^c^ (111–130)
H_ecc50_ (N·m)	131 ± 30^c^ (114–148)	139 ± 24^c^ (127–152)	127 ± 36^c^(107–145)	136 ± 21^c^ (124–147)
H_ecc40_ (N·m)	137 ± 31^c^ (120–154)	152 ± 27^a^ ^,^ ^c^ (138–167)	131 ± 45^c^ (108–155)	147 ± 24^a^ ^,^ ^c^ (134–160)
H_ecc30_ (N·m)	132 ± 39^d^ (113–155)	162 ± 35^a^ ^,^ ^d^ (143–180)	140 ± 49^d^ (113–166)	155 ± 30^a^ ^,^ ^d^ (139–171)
H_ecc20_ (N·m)	127 ± 43 (104–151)	164 ± 43^a^ (141–187)	142 ± 53 (113–170)	152 ± 42^a^ (130–174)
H_ecc10_ (N·m)	106 ± 49 (80–133)	146 ± 49^a^ (120–173)	125 ± 51 (98–152)	135 ± 42^a^ (113–157)

^a^ Significantly different from the eccentric mode, *p* < 0.05.

^b^ Significantly different from all angles, except 10°, *p* < 0.05.

^c^ Significantly different from all angles, except 10° and 20°, *p* < 0.05.

^d^ Significantly different from all angles, except 10°, 20° and 30°, *p* < 0.05.

**TABLE A3:** Mean ± standard deviation (95% confidence interval) for the average of the best two/three values of angle‐specific eccentric torque of the hamstrings (H_ecc_) collected in the eccentric and passive modes in the dominant (D) leg at 60°·s^−1^ (60) and 180°·s^−1^ (180).

	60		180	
	Eccentric	Passive	Eccentric	Passive
H_ecc100_ (N·m)	49 ± 9 (43–54)	23 ± 16^a^ (13–32)	45 ± 10 (39–52)	22 ± 11^a^ (15–29)
H_ecc90_ (N·m)	76 ± 16 (67–86)	52 ± 25^a^ (37–67)	87 ± 22 (74–101)	52 ± 22^a^ (39–65)
H_ecc80_ (N·m)	97 ± 19 (86–108)	80 ± 29^a^ (62–97)	101 ± 15 (92–110)	86 ± 22^a^ (72–99)
H_ecc70_ (N·m)	118 ± 19 (106–129)	100 ± 29^a^ (82–118)	111 ± 20 (99–124)	110 ± 23^a^ (96–125)
H_ecc60_ (N·m)	135 ± 22 (122–149)	120 ± 28 (104–137)	128 ± 22 (115–142)	130 ± 25 (115–145)
H_ecc50_ (N·m)	149 ± 26 (134–165)	138 ± 27 (122–154)	144 ± 25 (129–159)	140 ± 27 (123–156)
H_ecc40_ (N·m)	161 ± 31 (142–179)	152 ± 25 (137–168)	156 ± 28 (139–173)	156 ± 29 (139–174)
H_ecc30_ (N·m)	172 ± 29 (154–190)	164 ± 27 (148–180)	165 ± 33 (145–185)	167 ± 33 (147–187)
H_ecc20_ (N·m)	171 ± 37 (149–194)	169 ± 39 (145–192)	157 ± 49 (127–187)	161 ± 46 (134–189)
H_ecc10_ (N·m)	149 ± 42 (123–174)	155 ± 42 (130–180)	134 ± 45 (106–161)	134 ± 47 (106–163)

^a^ Significantly different from the eccentric mode, *p* < 0.05.
